# Atypical resting-state gamma band trajectory in adult attention deficit/hyperactivity disorder

**DOI:** 10.1007/s00702-021-02368-2

**Published:** 2021-06-23

**Authors:** László Tombor, Brigitta Kakuszi, Szilvia Papp, János Réthelyi, István Bitter, Pál Czobor

**Affiliations:** Department of Psychiatry and Psychotherapy, Semmelweis University, Balassa utca 6., Budapest, U1083 Hungary

**Keywords:** Adult ADHD, QEEG, Resting-state, Gamma power, Trajectory

## Abstract

Decreased gamma activity has been reported both in children and adults with attention deficit/hyperactivity disorder (ADHD). However, while ADHD is a lifelong neurodevelopmental disorder, our insight into the associations of spontaneous gamma band activity with age is limited, especially in adults. Therefore, we conducted an explorative study to investigate trajectories of resting gamma activity in adult ADHD patients (*N* = 42) versus matched healthy controls (*N* = 59). We investigated the relationship of resting gamma activity (30–48 Hz) with age in four right hemispheric electrode clusters where diminished gamma power in ADHD had previously been demonstrated by our group. We found significant non-linear association between resting gamma power and age in the lower frequency gamma_1_ range (30–39 Hz) in ADHD as compared to controls in all investigated locations. Resting gamma_1_ increased with age and was significantly lower in ADHD than in control subjects from early adulthood. We found no significant association between gamma activity and age in the gamma_2_ range (39–48 Hz). Alterations of gamma band activity might reflect altered cortical network functioning in adult ADHD relative to controls. Our results reveal that abnormal gamma power is present at all ages, highlighting the lifelong nature of ADHD. Nonetheless, longitudinal studies are needed to confirm our results.

## Introduction

ADHD is the most prevalent childhood mental disorder, and is characterized by inappropriate inattention, hyperactivity and impulsivity compared to healthy peers. ADHD persists into adulthood with full diagnostic criteria, or with a partial remission and concomitant impairment in circa 50% of cases (Faraone et al. 2006). It has an estimated prevalence between 1 and 3% in adults (Bitter et al. 2010; Simon et al. 2009). Although the symptoms, clinical characteristics, and neurocognitive profile of ADHD change over time, there is still a lack of knowledge about the lifespan aspects of the electrophysiological alterations of the disorder (Franke 2018).

Abnormalities of resting EEG activity in Attention Deficit/Hyperactivity Disorder (ADHD) have been reported in several previous publications (for a review see Loo and Barkley 2005; McVoy et al. 2019). Electrophysiological signals undergo a maturation process from newborn to adult ages (Clarke et al. 2001a; Gasser et al. 1988a, b; Scraggs 2012). Due to the significance of maturational processes during childhood, prior publications on age-related EEG changes in ADHD mostly focused also on childhood or the adolescence. The results were often interpreted in the context of the delayed maturation/maturational lag model (Bresnahan et al. 1999; Clarke et al. 2001a, b; El-Sayed et al. 2003; Loo and Barkley 2005), namely that ADHD patients’ behavior and their EEG measures would be normal in younger ages. On the other hand, the majority of previous studies also failed to confirm the existence of ADHD-specific developmental trajectories. In fact, electrophysiological maturation in ADHD was found to be quite similar to the maturation occurring in healthy controls (i.e., general decrease in absolute power measures) until early adulthood (Bresnahan et al. 1999; Clarke et al. 2019; Giertuga et al. 2017; Liechti et al. 2013; Poil 2014), and only few studies reported possible ADHD-specific alterations in electrophysiological development (Poil et al. 2014). The most consistent finding of previous research is that the elevated theta power, often prominent in children, is still present in ADHD across older age groups (Bresnahan et al. 1999; Clarke et al. 2019). In general, the main focus of prior works was the comparison of electrophysiological changes between (two) developmental stages (i.e., *from child- into adulthood*) which does not allow for an investigation of the EEG trajectories throughout the entire adult lifespan. Specifically, prior investigations were based on non-overlapping age cohorts, and the adult cohort was not sub-divided into adult age groups, which precludes conclusions about fine-grained changes in adulthood. Thus, our insight into the underlying electrophysiological changes in adulthood is still limited.

Gamma band activity is related to higher-order cognitive processes that rely on extended cortical networks via its important role in the functional organization of these networks (Uhlhaas et al. 2009a; Uhlhaas and Singer 2011). Neural networks undergo significant maturation from child- to adulthood (Khan 2018; Rojas et al. 2006). Moreover, marked structural and functional changes of these networks have also been reported over the adult lifespan in healthy individuals, which is reflected by the associations of different network measures with age during normal aging, starting as early as middle-aged adulthood (Bagarinao 2019; Hou 2018; Madhyastha and Grabowski 2014; Siman-Tov et al. 2016; Spreng et al. 2016; Tomasi and Volkow 2012). Deviations from the normal trajectory might be present in the case of neuropsychiatric disorders (Kaufmann 2019; Michels 2017; Onoda et al. 2012; Uhlhaas and Singer 2011) which contribute to the network dysfunction hypothesis of their pathophysiology.

Resting gamma band activity is decreased in childhood ADHD in comparison to healthy peers (Barry et al. 2009, 2010; Dupuy et al. 2014). In a previous publication, our group reported decreased resting EEG gamma band (30–48 Hz) activity in *adult* ADHD compared to healthy controls. Group differences exhibited right-lateralized scalp topography including an anterofrontal (Cluster A), a right central (Cluster B) and an extended right centro–parieto–occipital (Cluster C) distribution in the gamma_1_ band (30–39 Hz) and a smaller, right centroparietal (Cluster D) localization in the gamma_2_ band (39–48 Hz). Resting gamma power was inversely associated with ADHD symptom severity in both frequency ranges. (Tombor et al. 2018).

Regardless of the lifelong nature of ADHD, possible associations of EEG power measures and age in adulthood have rarely been addressed so far and none of the previous studies explored the developmental trajectories of resting-state gamma band in adult ADHD. It is also unknown whether the gamma band activity reduction found in cross sectional studies in ADHD persists to the same extent throughout the adult lifespan. Our aim was to conduct an exploratory study into the course of gamma power over time. Since gamma band activity is related to higher-order cognitive processes that are relevant in ADHD (e.g., attention functions Benasich et al. 2008; Bosman et al. 2014; Lenz et al. 2010; Prehn-Kristensen et al. 2015; Yordanova et al. 2000, 2002), and change with age in the disorder (Francx 2015; Thissen 2015; van Lieshout 2019), we hypothesized that gamma power trajectories in patients would be altered as compared to those found in healthy controls. Given that neurocognitive functions and gamma band EEG activity are strongly related, delineation of abnormal gamma trajectories in adult ADHD may ultimately contribute to our insight into the changes of the clinical presentation of the cognitive symptoms over time in the disorder.

## Methods and materials

### Participants

A total of one hundred and one subjects were included in the data analysis: 42 subjects with ADHD and 59 healthy controls, matched to the patients within ± 5 years of age. Of the 42 ADHD subjects, 25 were treatment naïve for ADHD, while 17 received methylphenidate (MPH) treatment. At the time of the study, only methylphenidate was available for adults with ADHD. Adult ADHD participants were recruited from the Adult ADHD Outpatient Clinic of the Department of Psychiatry and Psychotherapy, Semmelweis University, Budapest, Hungary. Controls were recruited from the local community, clinical staff and their relatives.

The inclusion criterion for the patient group was the diagnosis of ADHD based on a detailed clinical interview conducted in three steps as described by our group earlier (Simon et al. 2009): (1) structured interview for assessing the DSM-IV ADHD symptoms currently and retrospectively in childhood; (2) semi-structured and open interviews assessing background information, developmental data, functional impairment, psychiatric co-morbidity; and (3) medical history data obtained from close family members. Healthy control subjects were included if they had a negative history of any mental or neurologic disorders. Exclusion criteria for all participants included a history of severe neurological or somatic disorder or severe head trauma.

All participants gave written informed consent. The study was approved by the Institutional Research Ethics Committee of Semmelweis University and was conducted according to the Declaration of Helsinki.

### Measures

The Conners’ Adult ADHD Rating Scale (Conners 1998) (CAARS, 66-item self-reported, long version) was applied for the assessment of symptom severity. The CAARS has four subscales (Inattention/Memory problems, Hyperactivity/Restlessness, Impulsivity/Emotional problems and Problems with Self-concept) covering different symptoms associated with the disorder. As inattention, hyperactivity and impulsivity are considered as the core symptoms of ADHD (Arns et al. 2009), a Core Symptom Total Score was calculated in both study groups by the summation of Inattention/Memory problems, Hyperactivity/Restlessness and Impulsivity/Emotional subscale scores.

### EEG procedure and processing

EEG was recorded using a 128-channel Biosemi Active Two System (Biosemi, Amsterdam, Netherlands) in a sound-attenuated room. Participants were not allowed to take caffeine or to smoke two hours prior to EEG-recording. Participants were instructed to sit calmly with eyes open during the four minutes long resting-state EEG-recording. Electrooculogram (EOG) was recorded using two electrodes placed over and below the outer canthi. EEG was digitized with a sampling rate of 1024 Hz. Preprocessing was performed using EMSE Suite (Source Signal Imaging, San Diego, CA, USA). Data were band-pass filtered between 0.5 and 70 Hz and notch filtered at 50 Hz. For the ocular artifact removal, we used a spatial filtering approach, the Thin Plate Spline interpolation method described by Bookstein et al. (Bookstein 1989), and implemented in the Electro-Magnetic Source-Signal Estimation (EMSE) and imaging software (Pflieger 2001). This approach uses the topographical distribution of bioelectric signals from the EOG and EEG channels, respectively, to design a spatial filter in order to separate and project the EEG signal and the ocular artifacts into two orthogonal subspaces, and thereby removing the artifacts. In addition to the automatic artifact rejection, EEG recordings were also manually inspected, and gross artifacts including major eye movements were marked and removed from the analysis.

After preprocessing all EEGs were visually inspected and epochs containing artifacts were removed from further processing. Channels containing artifacts were marked and removed from analysis, and their data was replaced with interpolated voltage data. Artifact free, 2500 ms long epochs were used for Fourier transformation. Similar to prior studies (Barry et al. 2010; Dupuy et al. 2014), we focused on the low-gamma frequency band, ranging between 30 and 48 Hz. The target band was divided into two symmetrical frequency bins, 30–39 Hz and 39–48 Hz henceforth gamma_1_ and gamma_2_. Power spectrum data were further processed in second level analyses.

### Data analysis

For the comparison of demographic and clinical variables, Chi-squared tests were used for categorical and ANOVA for continuous variables.

For the analysis of between-group EEG power differences by age, our analyses focused on those electrode clusters in which our group previously reported significant differences between ADHD and controls in resting gamma band power (Tombor et al. 2018). Root-transformed gamma activities of each electrode in the predefined clusters had been averaged together to use a cluster-based statistical approach.

We used ANCOVA as principal statistical model for this investigation, as it represents a combination of the methods of regression and the analyses of variance, unifying the advantages of the two approaches in the context of a single general linear model as described in prior literature (Dunn and Clark 1974; Milliken and Johnson 2009). In particular, similar to ANOVA, it makes allowance for the use of categorical variables (such as study group in our investigation); and similar to regression, it allows for the use of continuous regressor variables (such as age in the current study). Resting gamma_1_ (30–39 Hz) and gamma_2_ (39–48 Hz) power were applied as dependent variables in separate analyses. Study group (ADHD vs. healthy controls) served as independent variable. Age was used as a continuous regressor. To investigate potential non-linear changes, age was included in the analysis both as a linear and a quadratic term. Interactions between study group and age (both in linear and quadratic terms) were also included in the analysis. Gender, treatment with MPH and overall symptom severity (Core Symptom Total Score) served as covariates.

Using age as continuous variable in the ANCOVA model, we computed the least-squares mean (LS-mean) estimates of gamma activity at every five year in the age range from 18 to 58 years for the purpose of visualization of the gamma trajectories separately in each study group for each electrode cluster. For the purpose of the computation of the LS-mean estimates and their standard errors, we used the linear and quadratic coefficients of age regression that reached significance in the statistical model.

To correct for multiple testing, in post-hoc comparisons performed with pairwise t-tests alpha was adjusted using Hochberg’s method (Hochberg 1988). All statistical analyses were performed with the SAS 9.4 version (SAS Institute Inc., Cary, NC, USA).

## Results

### Demographics and clinical characteristics

The summary of basic demographic and symptom severity characteristics is provided in Table 1. Age, gender and level of education were not significantly different between the study groups. Approximately three-quarters of the sample consisted of males, and mean age was slightly above 30 years. Median age was 27 years in the whole sample. Since a low proportion of younger and older study subjects might affect the results, we examined the frequency distribution of the study sample by classifying the subjects into three age groups: a group of younger (< 25-years old), an intermediate (25–35 years) and an older (> 35 years) age group. Our results (see Supplementary Table1 for details) indicated no statistically significant or clinically relevant difference between the age distribution of subjects in the ADHD and controls group (Chi-squared = 0.2354, *df* = 2, *p* = 0.89). Furthermore, the results showed that approximately 28.6% and 26.2% of the ADHD subjects fell, respectively, in the younger and older age groups (i.e., a total of 54.8% were outside the intermediate age range). The analogous numbers for the control subjects were 30.5% for the younger (< 25 years) and 22.0% for the older (> 35 years) subjects (with 52.5% being outside the intermediate age range).

**Table 1:** Basic demographics and clinical characteristics

Characteristic	ADHD (*N* = 42)	Control (*N* = 59)	*F*/*Z*	*p*
Age, years (Mean, SD)	30.92 (10.77)	30.88 (11.03)	0.0^a^	0.98
Median age, years (IQR^c^, Min–max)	28 (22–36, 18–57)	27 (24–33, 19–58)	0.64^b^	0.25
Conners’ Adult ADHD Rating Scale				
Core Symptom Total Score (mean, SD)	64.54 (13.77)	29.11 (14.83)	141.31^a^	< 0.0001
Hyperactivity/restlessness (mean, SD)	20.87 (6.68)	10.82 (5.99)	59.22^a^	< 0.0001
Inattention/memory problems (mean, SD)	24.91 (6.18)	9.87 (6.89)	121.05^a^	< 0.0001
Impulsivity/emotional problems (mean, SD)	18.75 (6.94)	8.4 (5.21)	68.97^a^	< 0.0001
Problems with self-concept (mean, SD)	10.36 (5.3)	4.49 (4.24)	35.92^a^	< 0.0001

^a^ANOVA, *F*

^b^Median test, *Z*

^c^*IQR* interquartile range

ADHD showed significantly higher overall symptom severity measured by the Core Symptom Total Score compared to controls. All four CAARS symptom factors were significantly higher in the ADHD group as compared to healthy controls (Table 1).

### Differences in gamma band activity by group and age

#### Gamma_1_

**Fig. 1:**
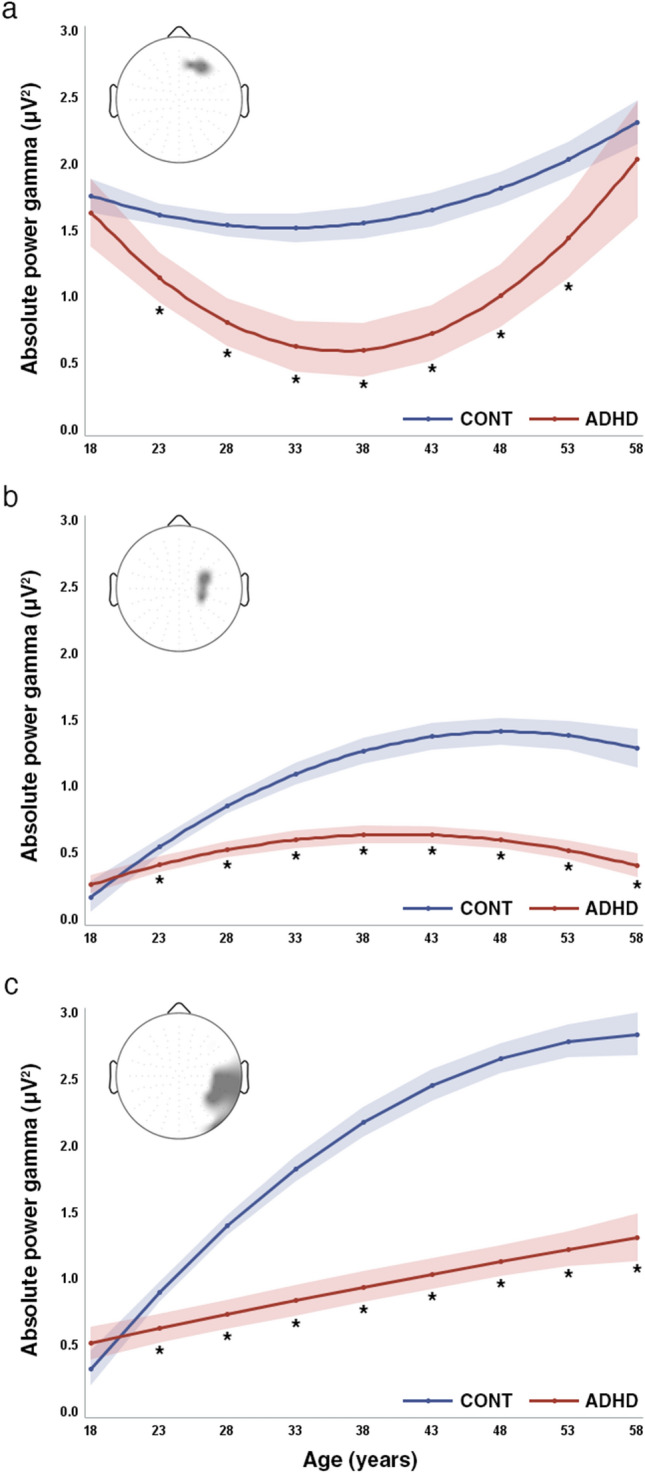
Resting-state gamma_1_ (30–39 Hz) power trajectory curves in the ADHD and healthy control groups. The scalp map embedded in the graph area in the upper left corner represents the scalp location of those leads where significant resting-state gamma_1_ power decrease has been identified in our previous investigation in ADHD relative to controls. Gamma_1_ power trajectories are depicted in the antero-frontal (**a**), in the right central (**b**) and the right centroparietal regions (**c**). **a** Shows a curvilinear trajectory for both ADHD and healthy control subjects. Faster reduction of gamma_1_ power is seen in ADHD than in controls, with significantly lower gamma_1_ power in patients starting from the early middle-ages. The difference diminishes, even though remains present until later adulthood. **b**, **c** Indicate that ADHD and healthy control subjects have non-linear increase of resting gamma_1_ in both scalp regions. The initially slightly higher resting gamma_1_ power at the youngest adult age diminishes. Then, during older ages, significantly lower gamma_1_ power and smaller increase of gamma activity can be found in ADHD than in controls, with the greatest difference in the older ages. At the end of the investigated age range, a slight decrease of gamma_1_ power is seen in both groups but differences remain significant. The red line represents ADHD patients, while the blue line represents healthy controls in all three panels. Shaded bands represent the 95% confidence limits for each curve. Asterisks (*) indicate significant post-hoc comparisons between the ADHD and control groups at the given age. The filled circles represent the least-squares mean (LS-mean) estimates of gamma activity at every five year in the age range from 18 to 58 years. For the purpose of the computation of the LS-mean estimates and their standard errors, we used the linear and quadratic coefficients of age regressions equations from the ANCOVA model. The interpolation of the trajectory point was based on the regression coefficients from the ANCOVA analysis

In the gamma_1_ band (30–39 Hz), the analysis adjusted for symptom severity and treatment indicated a main effect of group [*F*_(group)_ = 11.43, *p* = 0.0009], interactions of linear and quadratic age effect with group [*F*_(linear)_ = 19.0, *p* < 0.0001; *F*_(quadratic)_ = 16.59, *p* < 0.0001] in the right antero-frontal cluster (Cluster A). MPH treatment status, used as covariate in the analysis, also obtained significance [*F*_(treatment)_ = 70.5 *p* < 0001; treatment with MPH was associated a 41% relative increase of gamma activity in the treated group compared to the untreated]. Both groups exhibited curvilinear developmental trajectories of gamma_1_ activity. First, gamma activity decreased until around 40 years, followed by a plateau and then by an increase until the late fifties. This increment resulted in higher gamma_1_ power in the oldest ages of the sample. The initial reduction of absolute gamma power was more pronounced in patients than in controls. Except for the youngest and oldest ages, ADHD had significantly lower resting gamma activity throughout the investigated age range (Fig. 1, panel a).

In the right central and extended right centroparietal clusters (i.e., Cluster B and C) after adjustment for symptom severity and treatment we found significant main effects for study group [*F*_(group]_ = 15.25, *p* = 0.0001, *F*_(group)_ = 43.28, *p* < 0.0001, respectively) as well as interactions for study group with age for both the linear and the quadratic functions [*F*_(linear)_ = 16.05, *p* < 0.0001; *F*_(quadratic)_ = 6.95, *p* = 0.0087, *F*_(linear)_ = 46.84, *p* < 0.0001; *F*_(quadratic)_ = 24.09, *p* < 0.0001, for Cluster B and C, respectively]. MPH treatment status also obtained significance for both Cluster B [*F*_(treatment)_ = 13.12 *p* = 0.0003] and Cluster C [*F*_(treatment)_ = 127.67 *p* < 0001], with a 7% and 26% relative increase of gamma power for Cluster B and C, respectively, in the MHP treated subjects). At the earliest adult ages (< 20 years), ADHD had slightly higher right central and right centroparietal gamma_1_ power than controls. The developmental curves of gamma_1_ in these regions exhibited a curvilinear increase in both study groups. Gamma_1_ power in older ages was higher than in younger ages in both areas. In the right central region (Cluster B) gamma_1_ activity reached a plateau during the late thirties and early forties in ADHD, unlike in controls where a similar plateauing was observed slightly later. Gamma_1_ power decreased in both study groups in the upper limits of the investigated age range. A curvilinear increase of gamma_1_ power was found in both study groups over time in the right centroparietal region with a plateauing trend more apparently in controls. Except for the youngest ages, gamma_1_ power was significantly lower in ADHD then in controls over time in both scalp regions.

#### Gamma_2_

In the gamma_2_ (39–48 Hz) frequency band, the analyses adjusted for the covariates including treatment and symptom severity did not yield a significant main effect for group, [*F*_(group)_ = 0.11, *p* = 0.73], or the interaction effect of study group with age with respect to the linear [*F*_(linear)_ = 1.4, *p* = 0.23] or quadratic functions [*F*_(quadratic)_ = 2.29, *p* = 0.13]. MPH treatment status, applied as covariate, obtained significance in the analysis [*F*_(treatment)_ = 17.43 *p* < 0001; MPH treated patients had an 8% relative increase of gamma activity relative to MPH naïve patients]. The developmental path of gamma_2_ power is depicted in Figure S1 in Supplementary material S1. Both study groups had a reduction of gamma_2_ power over time with a slight acceleration of the decrease in controls during the mid-fifties. There were no group differences detected in any age.

## Discussion

The main finding that we report in this paper is a different developmental path of resting gamma_1_ (30–39 Hz) power in adult ADHD compared to healthy controls. The course of the gamma_1_ trajectory curves was a curvilinear decrease in both study groups in the antero-frontal brain areas. Patients with ADHD had a more pronounced decline in early adulthood, and except for the lowest and highest ages, they also had significantly lower gamma_1_ power then control subjects at all ages. Furthermore, both ADHD and control subjects had a curvilinear developmental path with an increasing gamma activity in the right central and right centroparietal regions. In the right central region, there was a modest gamma_1_ power increase in early adulthood, with a slightly earlier plateauing in the patients. Except for the youngest ages (< 20 years of age), ADHD patients had significantly lower gamma_1_ power than controls over time. The aforementioned results were statistically significant after adjusting for treatment status with MPH and symptom severity. In case of the gamma_2_ (39–48 Hz) absolute power, no significant association with age was found. Gamma_2_ decreased over time in both ADHD and controls.

We note that our results showed no difference at early adulthood between the ADHD and healthy control subjects. Decreased gamma activity in child and adult ADHD has been reported in previous literature as an overall group effect, usually by averaging measurement data from subjects of an age range. The analyses typically did not include group with age interaction effects. Furthermore, it should be kept in mind that the most relevant resting EEG literature on gamma activity in ADHD so far was based on groups of children at a relatively young age (7–12 years Barry et al. 2009, 2010; Dupuy et al. 2014)). For the above reasons, it is unclear whether the gap with respect to the difference in gamma activity between children with ADHD and age-matched healthy controls diminishes for early adulthood. We think that further studies should specifically focus on the transition period from late adolescence to early adulthood to examine this possibility.

To our knowledge, resting gamma power changes with age have not been investigated previously in adult ADHD. Despite that ADHD is considered a lifelong, neurodevelopmental disorder, the potentially altered late course of its neurobiological correlates, e.g., resting EEG measures or evoked potentials remain poorly addressed.

Previous publications typically report a decrease of EEG power measures within the traditional frequency bands (< 30 Hz) until early adulthood, but often fail to confirm disease-specific alterations during electrophysiologic maturarion (Bresnahan et al. 1999; Clarke et al. 2019; Liechti et al. 2013). Poil et al. (2014), however, delineated resting EEG trajectories in ADHD, which they interpreted as a reflection of developmental change characteristic for the disorder. Specifically, they found that beta power decreased during development and that the reduction was localized to small frontocentral and occipital regions in patients, unlike in controls where the decrease was global. Even though the authors did not discuss specific gamma power trajectories from childhood to early adulthood, they found a non-significant numerical decrease of gamma power with age.

Our findings indicate that the adult development paths of resting gamma power show significant decrease in ADHD as compared to control subjects. It is conceivable that these findings are associated with structural abnormalities and alterations of typical cortical development reported in the literature for ADHD (Proal 2011). Nonetheless, due to the scarce data on the normal development of gamma power in adulthood and its possible alterations in different neuropsychiatric disorders, it would be premature to conclude that our results are a reflection of these neurodevelopmental changes and abnormalities, and are specific to ADHD.

Tierney et al. (2013) investigated the age-related changes of resting gamma power in a healthy cohort aged 3–38 years. They reported a linear decrease of log transformed global gamma power (31–50 Hz) with age. Our results about the initial part of the developmental trajectory in younger adults in the antero-frontal leads are consistent with the decrease reported by Tierney et al.; It is of note, however, that our study investigated a broader age range, and was able to delineate the course of the trajectory curves at higher ages. Furthermore, instead of averaging the data globally for all recording channels, we conducted analyses for specific scalp regions. Thereby, we were able to detect different trajectory curves for different scalp regions and found that only the anterior findings from our study manifested the pattern described by Tierney et al. (based on the analyses of the pooled aggregate from all EEG channels in their study), suggesting a topographic specificity of the findings.

Specifically, with respect to scalp topography, our results indicate that the course of gamma power trajectories for both study groups in the antero-frontal regions is different than those found in the right central and right centroparietal regions. To interpret this finding, we should take into consideration that our study sample consisted of adults from 18 to 58 years of age. Such a broad age cohort has rarely been used in the ADHD literature. It is conceivable that the course of development in the antero-frontal leads might arise partly from the delayed maturation in ADHD and partly from early signs of normal cortical aging in controls which might not (yet) be present in the central and extended right centroparietal regions.

Early signs of cortical thinning, characteristic for the normal cortical aging, are already present in middle-aged healthy individuals in the prefrontal, frontal and, to a lesser extent, in the parietal heteromodal association cortices (Hoogman et al. 2017; McGinnis et al. 2011; Raz 2005). These cortical areas, the most vulnerable for aging, exhibit the greatest postnatal expanding during neural development, develop the latest and deteriorate the earliest during the lifespan (“last-in, first-out principle” (Raz and Rodrigue 2006)). The role of the “last-in, first-out” hypothesis has also been proposed in case of the P300 developmental trajectories in adult ADHD (Kakuszi et al. 2020). Besides, there is evidence from both cross sectional and longitudinal structural MRI studies that age-related reductions of the cortical structure and subcortical gray matter show regional variations in terms of extent and speed. Frontal and certain temporal cortical areas show faster reduction rates of volumetric data than other, more posterior regions like the occipital or insular cortices (Coupe et al. 2017; Storsve et al. 2014). Our results about the different trajectories seen in the anterior than more posterior leads might reflect these developmental differences. Nonetheless, it should be noted that EEG signals are not exclusively produced by the cerebral cortex but are summations of all electric signals produced by different cortical and subcortical sources, which assumes a more complex relationship between brain structure and electrophysiology.

Several publications report that spontaneous EEG band power and resting-state cortical networks are intercorrelated (de Pasquale et al. 2018; Mantini et al. 2007; Marino et al. 2019; Samogin et al. 2019). Gamma band activity plays an important role in the organization of cortical networks including intrinsic cortical networks operating at rest (Buzsaki and Wang 2012; Conner et al. 2011; Scheeringa 2011; Uhlhaas et al. 2009a, b; Werkle-Bergner et al. 2009). Dysfunctions of neural networks, especially the difficulty of switching between the default mode network and task-positive networks or imbalances between the dorsal and ventral attention networks might contribute to the pathophysiology of ADHD (Lin et al. 2015; McCarthy 2013; Sidlauskaite et al. 2016a, b; Sonuga-Barke and Castellanos 2007). Thus, our results that indicate different resting gamma_1_ trajectory in patients than in controls might reflect altered network operations throughout the entire lifespan in ADHD and might also underpin its lifelong course.

Our findings are consistent with results from previous studies which reported an increase in gamma power with stimulant treatment (even though not MPH specifically). Acute administration of amphetamine salts induced an increase of spontaneous MEG gamma activity (30–106 Hz) (Wilson et al. 2013) and 40 Hz gamma-band steady-state response (Wilson et al. 2012) in adult ADHD relative to controls. Similarly, Albrecht et al. found an increase of resting EEG gamma band (30–45 Hz) power (Albrecht et al. 2016), 40 Hz auditory steady state response (Albrecht et al. 2013) and wavelet transformed gamma activity during an oddball task (Albrecht et al. 2012) in a pharmaco-EEG study investigating the acute effects of dexamphetamine in healthy volunteers compared to the off-medication condition. However, we should note that our data are cross sectional, and therefore, cannot demonstrate causal relationship with treatment.

There are several limitations of our study. First, we applied a cross-sectional study design to delineate the associations of gamma band power with age. Although a longitudinal study design may provide better understanding of age-related changes, it is rarely adopted in the relevant literature due to feasibility reasons. Nonetheless, longitudinal studies would be important to confirm our results. Second, the subjects in the two study groups were not uniformly distributed across the entire age range. While subjects in the critically important lower (< 25 years) and higher age (> 35 years) ranges were included in our sample, we think that future studies should use a more targeted sampling design to assure a uniform age distribution. Third, major developmental changes occur from childhood through adolescence to early adulthood both in healthy controls and in ADHD. Since our study focused on adults, it is not possible to draw conclusions about the developmental path of gamma power in the earliest years of adulthood (< 20 years) where partially different trends were observed than in other age groups (Barry et al. 2010; Dupuy et al. 2014; Tombor et al. 2018). Fourth, while 40–60% of ADHD patients completely or partially remit during adolescence, our results are limited to those subjects who meet full criteria for ADHD as adults. Further studies are therefore needed to shed more light on the relationship of resting gamma power with network functions and to elucidate gamma trajectories in ADHD remitters and/or in the broader phenotype including relatives of ADHD probands.

## Conclusions

There was a curvilinear/non-linear association between resting gamma power and age in adult ADHD. Resting gamma power increased over time in both study groups. The course of trajectory curves is similar in patients than in controls, and ADHD patients had significantly lower resting gamma_1_ power (30–39 Hz) than controls across most of the age range in our sample. The maximum of the differences in the antero-frontal region was observed during the middle-age with a diminution of differences in later ages. In case of the central and centroparietal regions, gamma activity differences increased with time. The results suggest that diminished resting gamma activity is stable over time in adult ADHD, and that different developmental paths might be present over the frontal and centroparietal cortices. In the framework of the dysfunctional network hypothesis, these results might reflect alterations of cortical organization, neural network structure and dynamics during late neurodevelopment and early aging in ADHD, as it is considered both a neurodevelopmental and a lifelong disorder. Our results contribute to the literature on the lifespan course of adult ADHD, underline its lifelong nature and might help to gain insight into the age-related changes of potential biomarkers for use in clinical practice.
